# Novel Pathogenic Sequence Variants in *NR2E3* and Clinical Findings in Three Patients

**DOI:** 10.3390/genes11111288

**Published:** 2020-10-29

**Authors:** Saoud Al-khuzaei, Suzanne Broadgate, Stephanie Halford, Jasleen K. Jolly, Morag Shanks, Penny Clouston, Susan M. Downes

**Affiliations:** 1 Oxford Eye Hospital, John Radcliffe Hospital, Oxford University Hospitals NHS Foundation Trust, Oxford OX3 9DU, UK; saoud.al-khuzaei@stx.ox.ac.uk (S.A.-k.); jasleen.jolly@eye.ox.ac.uk (J.K.J.); 2 Nuffield Laboratory of Ophthalmology, Nuffield Department of Clinical Neuroscience, University of Oxford, Level 6 John Radcliffe Hospital, Headley Way, Oxford OX3 9DU, UK; suzanne.broadgate@ndcn.ox.ac.uk (S.B.); stephanie.halford@eye.ox.ac.uk (S.H.); 3 Oxford Medical Genetics Laboratory, Oxford University Hospitals NHS Foundation Trust, Oxford OX3 7LE, UK; Morag.Shanks@ouh.nhs.uk (M.S.); Penny.Clouston@ouh.nhs.uk (P.C.)

**Keywords:** NR2E3, inherited retinal degeneration, retinal dystrophy, enhanced S-cone syndrome, autosomal recessive and autosomal dominant retinitis pigmentosa, Goldmann–Favre syndrome, pigmentary clumping, ellipsoid zone

## Abstract

A retrospective review of the clinical records of patients seen at the Oxford Eye Hospital identified as having *NR2E3* mutations was performed. The data included symptoms, best-corrected visual acuity, multimodal retinal imaging, visual fields and electrophysiology testing. Three participants were identified with biallelic *NR2E3* pathogenic sequence variants detected using a targeted NGS gene panel, two of which were novel. Participant I was a Nepalese male aged 68 years, and participants II and III were white Caucasian females aged 69 and 10 years old, respectively. All three had childhood onset nyctalopia, a progressive decrease in central vision, and visual field loss. Patients I and III had photopsia, patient II had photosensitivity and patient III also had photophobia. Visual acuities in patients I and II were preserved even into the seventh decade, with the worst visual acuity measured at 6/36. Visual field constriction was severe in participant I, less so in II, and fields were full to bright targets targets in participant III. Electrophysiology testing in all three demonstrated loss of rod function. The three patients share some of the typical distinctive features of *NR2E3* retinopathies, as well as a novel clinical observation of foveal ellipsoid thickening.

## 1. Introduction

Inherited retinal dystrophies are a heterogeneous group of diseases with varying degrees of severity, age at onset and inheritance patterns. Retinal dystrophies associated with the gene *NR2E3* (nuclear receptor subfamily, 2 group, E member 3) can be inherited as either autosomal recessive or dominant. *NR2E3* retinal dystrophies include enhanced S-cone syndrome (ESCS) (ESCS, OMIM#268100) [1], a rare autosomal recessive condition, first described by Marmor et al. in 1990 [2]. Fundoscopy typically shows nummular pigmentary deposition at the level of the retinal pigment epithelium external to the vascular arcades with macular changes often associated with intra-retinal cysts [3,4]. Histopathologic analysis of a post-mortem retina affected by ESCS showed a highly degenerate and disorganised retina characterised by the absence of rods, and twice the number of expected cones. Intermixed cones are observed to be densely packed with the inner retinal neurons [5]. By using adaptive optics imaging in *NR2E3* retinas, a higher than normal density of cones has been shown to be present in the perifoveal retina, with smaller outer segment cone diameters [6]. Analysis of electroretinograms in patients with ECSC by Hood et al. supports the observation that there are many more S-cones than in a normal retina [7].

ESCS typically presents with an abnormally large S-cone response in the majority of patients with an associated undetectable rod-specific response. The relative increase in S-cone function compared to the L and M cone function is pathognomonic, and is not affected by disease severity [8,9]. Standard single flashes under both scotopic and photopic responses tend to be attenuated and delayed and of similar amplitude under both light conditions [3,8]. The 30 Hz flicker response displays a delayed peak time and reduced amplitude and the pattern electroretinogram (PERG) varies from normal to undetectable [3]. These changes are present from a young age, and are slowly progressive with increasing disease severity [10]; thus, ESCS electrophysiological features are very distinctive [3,8,10].

**Figure 1:**
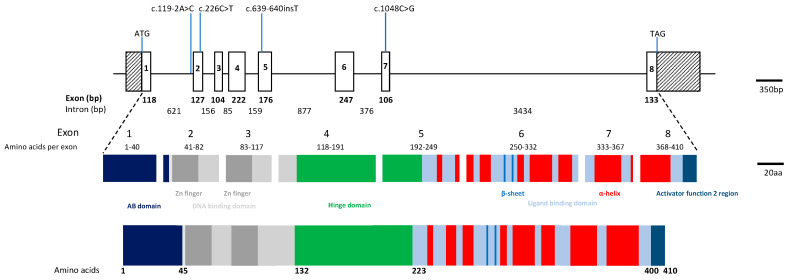
Schematic diagram of *NR2E3* genomic locus, structure of the encoded protein and location of identified mutations. The top panel shows the *NR2E3* gene on 15q23 which consists of 8 exons and spans approximately 7.7 kb of genomic DNA. Exons are shown as boxes and introns as lines; all are to scale. The middle panel shows which domains each exon contributes to the protein structure. The gene encodes a predicted 410 amino acid protein which shows the characteristic structure of a nuclear hormone receptor (lower panel) and consists of 5 domains: the N-terminal A/B domain (regions 1 and 2, shown in dark blue), a DNA binding domain (light grey) which contains 2 Zn-fingers (region 3, dark grey), a hinge region (region 4, green) and the ligand binding domain at the C-terminal end (region 5, light blue). The position of the β-sheets, α-helices and the activator function region 2 are also shown (in red, blue and darker blue, respectively). The positions of the mutations described in this report are also shown. Figure adapted from Mollena et al. [22].

Two other conditions have also been associated with autosomal recessive *NR2E3* mutations, Goldmann–Favre Syndrome (GFS), first described in the 1950s [11], and clumped pigmentary retinal degeneration (CPRD) [12]. GFS manifests as night blindness or a progressive decrease in visual acuity in the first decade of life [13,14,15]. Typical features include hyper-pigmented Retinal Pigment Epithelium (RPE) clumps accumulating along the retinal vascular arcades, areas of choroidal atrophy, a cystoid or schitic fovea, with central and/or peripheral retinoschisis, and vitreous degeneration. CPRD typically shows clinically visible clumped pigmentation in the mid-peripheral fundus in all four quadrants and is associated with minimal evidence of bone spicule pigmentation [16]. More recently, it has been suggested that GFS and clumped pigmentary retinopathy associated with *NR2E3* mutations could be considered part of the expanded phenotype of ESCS; both conditions share similar electrophysiological features to ESCS [8,17,18,19,20]. The *NR2E3* gene was identified by Kobayashi and colleagues in 1999 it is located on chromosome 15q23, comprises eight exons, spans approximately 7.7kb of genomic DNA and encodes a 410-amino acid protein (Figure 1) [21]. NR2E3 is a member of a large family of nuclear receptor transcription factors and the protein shows the characteristic structure of a DNA-binding domain and a putative ligand-binding region separated by a hinge region (Figure 1) [21,22]. It is expressed in the outer nuclear layer of the retina [1], and is found in both developing and adult rod photoreceptors [5,23,24]. *NR2E3* acts as a transcriptional regulator and has been shown to activate rod-specific genes as well as repressing cone-specific genes acting in tandem with other retinal transcription factors including cone-rod *otx*-like photoreceptor homeobox transcription factor (*CRX*) and neural retina leucine zipper transcription factor (*NRL*) [24,25,26,27,28].

In this study, we describe the detailed clinical features in three patients with *NR2E3* mutations and report two novel pathogenic sequence variants.

## 2. Materials and Methods

Three of 4 patients identified to have *NR2E3* pathogenic variants by the Oxford Molecular Genetics Laboratory were included in this study; the fourth patient had been previously reported by Cehajic-Kapentanovic et al. [29]. This study was conducted in accordance with the Declaration of Helsinki with Ethics approval obtained from the local research ethics committee (reference 08/H0302/96) and informed written consent was obtained from all patients.

### 2.1. Literature Search

A literature search was performed to identify reports of *NR2E3* pathogenic sequence variants (Supplementary Table S1).

### 2.2. Clinical Data

The clinical data of 3 patients (I, II, III) identified as having 2 *NR2E3* variants by the clinical laboratory were reviewed. Data extracted included age, sex, family history and pedigrees, ethnicity, history of symptom onset and any associated conditions, clinical assessment included best corrected visual acuity, slit-lamp biomicroscopy, pseudo-colour fundus imaging using a wide field scanning laser ophthalmoscope (optomap; Optos), short-wavelength fundus autofluorescence (AF) (Spectralis; Heidelberg Engineering, Heidelberg, Germany), and spectral-domain optical coherence tomography (SD-OCT; Spectralis, Heidelberg Engineering, Heidelberg, Germany); Goldmann visual fields and electrodiagnostic testing were obtained from the electronic and clinical notes. The Heidelberg software was used for retinal segmentation of the ganglion cell layer (GC) and the inner plexiform layer (IPL) which were then used to measure the thickness of the GCIPL thickness in the nasal temporal areas and inferior part of the central macula, which are summarised in Supplementary Table S2. Electroretinography was performed in accordance with the standards of the International Society of Electrophysiology of Vision (ISCEV) using DTL fibre electrodes and an impedance <5 kOhms in pupils dilated with 1% tropicamide [30].

### 2.3. Molecular Genetic Analysis

Genetic analysis was performed at the Oxford Regional Genetics Laboratory, Oxford University Hospitals NHS Foundation Trust as part of the patient’s routine care, as described previously [31]. Samples were prepared using Agilent’s HaloPlex™ Target Enrichment system for a 111-gene panel, followed by next generation sequencing on Illumina MiSeq Personal Sequencer. All findings were validated by Sanger sequencing. In silico analysis using 3 different prediction methods were used to determine the deleteriousness of the variants, Polyphen2 (available at http://genetics.bwh.harvard.edu/pph2/, accessed on 1 June 2020) [32], Sorting Intolerant from Tolerance (SIFT) (available at http://sift.jcvi.org/, accessed on 1 June 2020) [33] and Mutation Taster (available at http://www.mutationtaster.org/, accessed on 1 June 2020) [34], on the variants identified (Table 1).

## 3. Results

**Figure 2:**
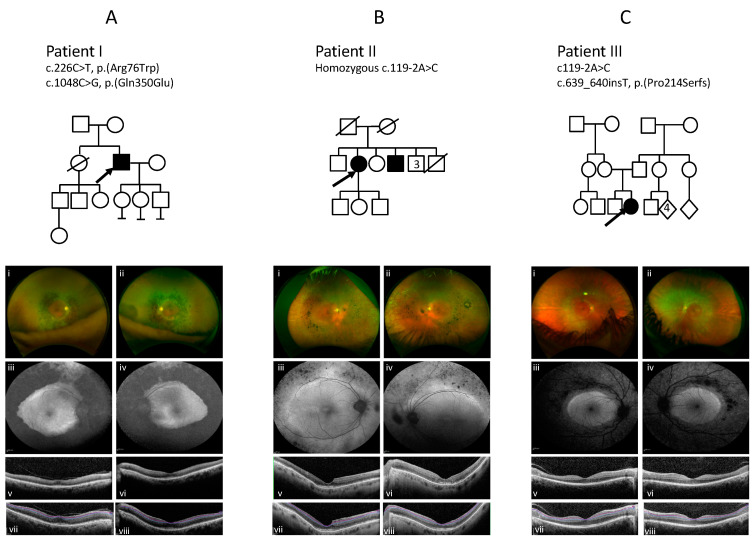
Family trees and colour imaging, fundus autofluorescence (FAF), and spectral domain optical coherence tomography (SD-OCT) imaging of patients I, II and III. The top panel shows the pedigrees of each family, the arrow indicates the proband. (**A**) Imaging of Patient I showing a dense concentric band of pigment and atrophy, extending from the arcades into the mid peripheral retina (**i**,**ii**); increased autofluorescence signal at both maculae (**iii**,**iv**), and central preservation of the retina on optical coherence tomography (**v**,**vi**). (**B**) Imaging of Patient II showing concentric pigment clumping in the mid peripheral retina that is more pronounced in the temporal and nasal regions (**i**,**ii**); mildly increased AF at both maculae and decreased AF extending from the vascular arcades into the midperiphery (**iii**,**iv**); significant focal thickening at the fovea in the ellipsoid zone (EZ) and, in the right eye, an incipient lamellar hole (**v**,**vi**). (**C**) Imaging of patient III shows a concentric band of atrophy, pigment, and white dots extending from the arcades into the mid peripheral retina (**i**,**ii**); with an increased autofluorescence signal at both maculae (**iii**,**iv**), and central preservation of the retina on optical coherence tomography (**v**,**vi**). (**vii**–**viii**) in each panel are OCT images with segmentation highlighting the ganglion cell layer in purple and the inner plexiform layer in blue (see Supplementary Table S2 for measurements).

*NR2E3* variants were identified in three patients, from unrelated families, who had been diagnosed with retinal dystrophy (Table 1). The pedigrees of the families and imaging are presented in Figure 2 and the clinical findings are summarised in Table 2.

### 3.1. Patient I

**Figure 3:**
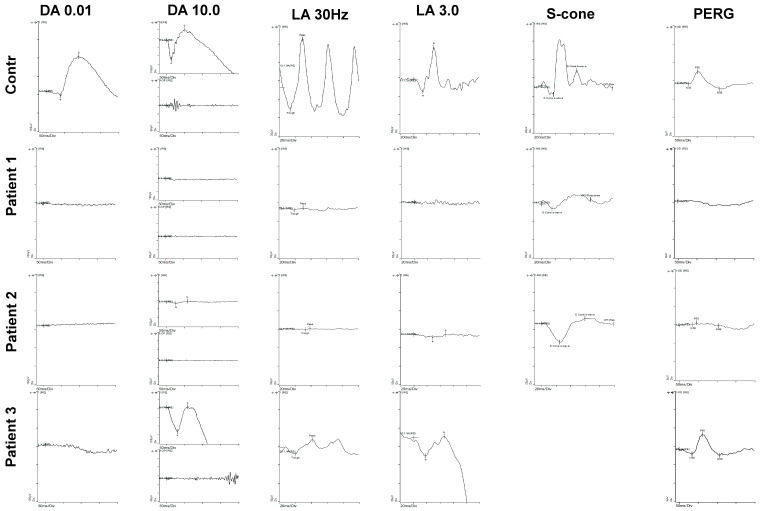
Electrophysiology results for patients compared to a control eye. **Patient 1** has widespread loss in all systems with residual S-cone response. **Patient 2** has extinguished rod, reduced cone but large S-cone responses. **Patient 3** has reduced responses affecting rods more than cones. DA 0.01 is the dark-adapted rod response, and DA 10 is the dark adapted maximal response. At DA 10, both the single flash response and oscillatory potentials are shown. LA 30 Hz is light adapted 30 Hz flicker response and LA 3 is the light adapted standard flash as per International Society of Electrophysiology of Vision (ISCEV) guidelines. The S-cone response isolates the S-cone response using a blue flash. The pattern electroretinogram (PERG) reflects macular function.

A 70-year-old Nepalese male with nyctalopia since his 20s underwent a review at 66 years of age reporting the onset of flashing lights, increasing constriction of his visual fields and blurred central vision. Examination revealed visual acuities of 6/19 OD and 6/24 OS. Goldmann visual fields (GVF) showed less than five degrees of field preservation. He had a posterior subcapsular lens opacity OD. Fundoscopy through dilated pupils revealed a dense concentric band of pigment and atrophy extending from the arcades into the mid peripheral retina. Autofluorescence (AF) imaging showed a dense loss of AF signal extending from the arcades into the mid periphery consistent with the band of atrophy, with increased AF at both maculae extending to the nasal area. OCT showed central preservation of the retina, vitreomacular traction in OD, and focal thickening at the fovea of the ellipsoid zone (EZ) and loss temporally, but no oedema (Figure 2A). Electrophysiology testing demonstrated severe widespread rod, cone, macular and RPE dysfunction; however, S-cone testing revealed a residual response (Figure 3). Genetic testing identified a previously reported c.226C > T, p.(R76W) mutation [1,35] and a novel mutation c.1048C > G, p.(Q350E) in *NR2E3* (Figure 1).

### 3.2. Patient II

A 69-year-old white Caucasian female with a lifelong history of nyctalopia and abnormal colour vision since the age of 45 was initially diagnosed with retinal dystrophy at the age of 40 years. She had a previous medical history of diabetes and vitiligo. Her brother also has a diagnosis of Retinitis Pigmentosa (RP) and diabetes (but no other details were available). An examination revealed visual acuities of 6/36 + 1 OD and 6/36 OS. Anterior segment review showed bilateral early cataract, minimal epiretinal membranes (Figure 2B). Fundoscopy revealed concentric nummular clumped pigmentary deposition at the vascular arcades and extending into the mid periphery, which was more pronounced in the temporal and nasal areas with white spots in the far periphery. AF imaging showed mildly increased AF at the maculae and decreased AF extending from the vascular arcades into the midperiphery in a similar distribution to the clumped pigment seen on fundoscopy. The features on OCT imaging included significant thickening at the fovea and parafoveal region in the EZ and in the right eye an incipient lamellar hole (Figure 2B). Electrophysiology testing showed a pattern consistent with ESCS (Figure 3). The PERG was almost extinguished. Both rods and cones were grossly affected, with a similarly reduced and delayed response to standard flash under both scotopic and photopic conditions. The 30Hz flicker was markedly abnormal in the presence of a large S-cone response. Genetic testing identified that she was homozygous for the common *NR2E3* splicing variant, c.119-2A > C [1] (see Supplementary Table S1 for other references). Taken together, these findings are consistent with the pigmentary clumping subgroup of *NR2E3* phenotypes.

### 3.3. Patient III

A 16-year-old girl presented with symptoms at the age of 10 years; these included photophobia, photosensitivity, photopsia, and nyctalopia. Examination revealed visual acuities of 6/4 OD and 6/5 OS with refraction of +0.75/−0.75 × 30 OD and L +1.00/−1.00 × 140 OS. Fundoscopy revealed an atrophic concentric band with white punctate dots and minimal pigment extending from the vascular arcades to the mid-peripheral retina circumferential to the macula. Visual fields showed decreased sensitivity from 10 degrees to 40 degrees on Goldmann visual field testing. Colour vision testing with colour contrast sensitivity showed reduced protan thresholds and normal tritan responses. An increased (AF) signal was seen at the macula and extending to the nasal area. Optical coherence tomography (OCT) showed loss of the EZ in the area of the band, with preservation of the central retina (Figure 2C). The ERG results demonstrated extinguished rod-specific dim flash responses and reduced and delayed responses to standard flash under both photopic and scotopic conditions. Response to the 30 Hz flicker stimulus was abnormal but the PERG was relatively well preserved. Isolated S-cone responses were not measured. The marked reduction to rod stimuli and 30 Hz flicker with reduced responses to standard flash is consistent with an ESCS phenotype (Figure 3). Genetic molecular analysis showed compound heterozygosity for *NR2E3* c.119−2A > C [1] (see Supplementary Table S1 for other references) and a novel mutation c.639_640insT, p.(P214SfsX39). Segregation analysis in her parents identified the *NR2E3* variant c.639_640insT, p.(P214SfsX39) in her father, and the c.119-2A > C variant in her mother.

## 4. Discussion

Disease-causing mutations in *NR2E3* have been found to be located throughout the gene, but are predominantly found in the regions of the gene encoding the DNA binding domain (amino acids 45-131) and ligand binding domain (amino acids 223–410) of the NR2E3 protein (see Supplementary Table S1 and Figure 1). The most frequently reported mutations in *NR2E3* are c.119-2A > C, p.(V41AfsX23) and c.932G > A, p.(R311Q) (see Supplementary Table S1 for references).

The mechanisms underlying the differing retinal phenotypes associated with *NR2E3* variants remain unclear. However, there is a naturally occurring mouse model of *Nr2e3*, the *rd7* mouse, which has a 380 bp deletion of the coding region that removes amino acids 110-236, and generates a frameshift and premature stop codon. The protein that is produced is most likely subjected to nonsense-mediated decay. The equivalent region in the human sequence is between amino acid 117 and 249. This deletion would result in the loss of the 3′ end of the DNA binding domain, the entire hinge region and helixes 3 and 4 of the ligand binding domain (Figure 1). The *rd7* mouse, shows up to a twofold increase in S-cones [36]. Chen et al. showed that *Nr2e3* is expressed exclusively in rods in this mouse model, and that expression of *Nr2e3* is one of the earliest events in rod photoreceptor development and that it suppresses cone-specific genes [25]. The loss of *Nr2e3* also leads to the de-repression of cone specific genes in rods and Chen et al. suggest that the misexpression of many of these cone transcripts may have toxic consequences for the rod cells and can result in the degeneration of the rods that is seen in patients with *NR2E3* variants. However, Xie et al. did not identify an excess number of S-cones using *Nr2e3* knock out zebrafish, created using CRISPR technology [37]. They demonstrated both the normal development and number of cones, with no excess of S-cones, and showed that the L and M cones progressively degenerated. They also demonstrated that rod precursors failed to differentiate into rods; rod-specific genes were not expressed, and rod outer segments did not develop [37].

In our patient cohort, we identified two novel mutations in *NR2E3*: c.1048C > G, p.(Q350E) in patient I, and c.639_640insT, p.(P214SfsX39) in patient III. The previously reported mutations were as follows: c.226C > T, p.(R76W), was seen in patient I; c.119-2A > C was seen as homozygous in patient II and heterozygous in patient III (Table 1). All three patients showed signs of pigment clumping that extended in a band from the vascular arcades to the mid-periphery with a characteristic corresponding band of a decreased signal in AF extending from the vascular arcades to the mid periphery, which is consistent with the literature regarding patients with recessive *NR2E3* mutations [3,8,19,35,38,39,40,41]. An increased macular AF signal was also noted. White spots were seen in the periphery in patient II and in the band in patient III, but were smaller and less sparse than those described by Murro et al. [35]. The distribution of white deposits located within the band is consistent with the paediatric patients with *NR2E3* variants described by Cassiman et al. and Hull et al. [10,42]. These features are consistent with an early stage of the disease prior to the development of pigment clumping seen later in life. This is consistent with the observations made by Hull et al. who proposed that retinal disease secondary to *NR2E3* variants is progressive, and showed this in their paediatric population, describing a sequence of change from a normal fundus appearance to RPE mottling along the arcades, with development of white dots with subsequent deposition of nummular pigment [10]. Cassiman et al. reported a paediatric phenotype of *NR2E3* patients with submacular haemorrhage and preretinal fibrosis before the appearance of macular nummular pigmentary deposition at the level of the RPE [42]. Patient III showed no evidence of this. Moreover, none of the three patients reported here had cystoid or schitic changes at the macula, as has been described as a typical or common appearance in *NR2E3* recessive retinopathies [2,3]. None of the three patients showed a double or triple hyperfluorescent ring sign as reported by Escher et al. on AF imaging [43]. Notably, in patient II particularly, and also in patient I, a focal significant thickening of the ellipsoid layer at the fovea was observed (Figure 2A,B).

The electrophysiology testing in all the patients was consistent with an *NR2E3* retinopathy, two presenting with features of ESCS and one with RP characteristics. Patient I presented with severe general retinal degeneration. Interestingly, patients II and III showed extinguished responses to rod-specific dim flash, reduced to 30 Hz flicker, and reduced to standard flash in both in both photopic and scotopic conditions. These are in keeping with previous reports. The literature shows that the visual acuities in *NR2E3* retinopathies are variable; in these three patients, the visual acuities were relatively good. However, patients I and II had less than five degrees of visual field.

It is clear, upon reviewing all the previously reported studies (see Supplementary Table S1), that severity varies considerably in the retinopathies associated with *NR2E3*. This study, reporting 3 cases with variable age at onset, sex and ethnicity, is consistent with this finding. To date, no clear phenotype-genotype correlation has been identified in *NR2E3* retinopathies. Indeed, phenotypic appearance can vary even when patients have the same variant. For example, Bandah et al. described phenotypes associated with the c.119-2A > C variant ranging from minimal fundoscopic changes with retinal flecks and a preserved foveal structure in a 19-year-old, compared to a 16-year-old with patches of deep chorioretinal atrophy with macular involvement, and a 20-year-old with perimacular atrophy, classic pigment clumps beyond the arcades, and severe cystoid macular oedema [44]. Most previous studies have suggested that the c.119-2A > C variant causes the skipping of exon 2 and a premature termination codon in exon 3, leading to nonsense-mediated decay of the aberrant protein [44,45]. However, in a study by Bohrer et al. using patient induced Pluripotent Stem Cell (iPSCs), the authors suggest that a cryptic acceptor site in intron 1 is activated by the variant and a region of intron one is incorporated into the transcript as well as exon two, which would also result in a termination codon in exon 3 [46]. It has been suggested by Bandah et al. that, due to the mutation occurring in the second intronic base of the donor splice site, a variable amount of wild type protein can still be generated in patients with the c.119-2A > C variant [44]. Taken together, these data suggest that the c.119-2A > C variant is complex and may behave differently between patients. The presence of a variable amount of protein may explain the variability in the phenotype in patients harbouring this variant.

Two of the three patients described here carry the c.119-2A > C variant. Patient II is homozygous and Patient III carries the variant heterozygously in *trans* with a c.639_640insT variant that results in the frameshift p.(P214SX39). Patients I and II are older and it may have been expected that their phenotype would have been more severe due to prolonged disease progression, however, Patient III has quite severe disease at an early age. The variants in patient III are both frameshift mutations which are predicted to produce truncated proteins which will most likely be cleared by nonsense-mediated decay. Thus, the variant c.119-2A > C can result in a variable amount of protein and may, in this case, produce very little wild-type protein, leading to the severe phenotype.

Patient I harbours two missense variants, the previously reported p.(R76W) and the novel p.(Q350E), which are located in the DNA binding domain and the ligand binding domain, respectively. Previous studies have shown that the p.(R76W) variant results in non-specific DNA binding and the protein mis-localising to the cytoplasm [47]. The p.(Q350E) is novel to this study, but previous studies have investigated the nonsense mutation at this position p.(Q350X) [16,48]. The glutamine at position 350 is located in helix nine of the ligand binding domain, close to the dimer interface and p.(Q350X) protein has been shown to mis-localise in the cytoplasm [47].

There are many factors can influence the range of phenotypes seen in a disease including; epigenetic factors, environment, allelic heterogeneity and genetic modifiers [45,49,50]. The presence of as yet unidentified polymorphisms in other genes known to interact with *NR2E3* such as *NRL*, *CRX*, *NEUROD1*, *RORA* and *TRβ2* may also be influencing the phenotypic outcome [26].

## 5. Conclusions

*NR2E3* retinopathies are very rare, we describe three patients in whom *NR2E3* mutations have been identified, of which two are novel. The typical features previously described including a band of atrophic retinal pigment epithelium, white dots, and nummular pigment deposition were seen in our patients. We also identified a focal foveal thickening at the level of the EZ. This study shows that macular cystoid or schitic changes are not always present in *NR2E3* retinopathies. In view of the variable phenotypes, we propose that the three entities ESCS, CPRD and GFS are renamed *NR2E3* retinopathies, listing key features, which would include visual acuity, field measurements, the presence of oedema, and electrophysiological characterisation to help in determining prognosis. OCT imaging is becoming increasingly used as an outcome measure in retinal therapies to assess response to treatment; thus, characterising novel OCT features in specific diseases is very relevant.

Interestingly, a recent study of a mouse model has shown that delivery of *NR2E3* to retinal cells stabilises disease progression [50]. With potential treatment prospects in the pipeline, obtaining a genetic diagnosis and characterising the phenotype in depth is of increasing importance.

## Figures and Tables

**Table 1:** Summary of *NR2E3* variants identified.

Patient	Variant	Protein	Genotype	Position	Location	gnomAD MAF	PolyPhen2	SIFT	Original Reference
**I**	c.226C > T	p.R76W	Het	Exon 2	chr15: 72103930	0	D	D	[1]
	c.1048C > G	p.Q350E	Het	Exon 7	chr15: 72106406	0	D	D	Novel
**II**	c.119-2A > C	Splicing	Hom	Intron 1	chr15: 72103821	0.0005	n/a	n/a	[1]
**III**	c.119-2A > C	Splicing	Het	Intron 1	chr15: 72103821	0.0005	n/a	n/a	[1]
	c.639_640insT	p.P214SfsX39	Het	Exon 5	chr15: 72104744	0.000004	n/a	n/a	Novel

Chromosome position is based on build GRCh37/hg19; nucleotide and protein numbering is based on *NR2E3* transcript NM_014249.3; Genome Aggregation Database, gnomAD; MAF, minor allele frequency; gnomAD, Polyphen, and SIFT were accessed on 1 September 2019. Polyphen predictions range from zero to one and variants are appraised qualitatively as benign (B) (0.00–0.15), possibly damaging (P) (0.16–0.85), or probably damaging (D) (0.86–1.00). SIFT results are reported to be tolerant (T) if tolerance index > 0.05 or intolerant (damaging (D)) if tolerance ≤0.05. Novel variants identified in this study are shaded.

**Table 2:** Clinical information.

Patient	Gender	Ethnic Origin	First Symptoms (and Age at Diagnosis)	Age of Last Examination (Years)	Symptoms at Last Review & Presence of Cataract	Best Corrected VA	Fundus	OCT	Autofluorescence	Visual Field	ERG	Variant
**I**	M	Nepalese	Night blindness since childhood diagnosed (20)	66	Nyctalopia, blurred central vision, photopsia Cataract bilateral: posterior subcapsular	6/18 RE 6/24 LE	Ring of dense pigment with atrophy surrounding and including the arcades extending to the mid periphery; sparing maculae and peripheral retinas	Central preservation of the retina with loss of the EZ temporal to macula, vitreomacular traction OD Thickened EZ/IZ	Annulus of decreased signal consistent with coalescing atrophy extending from and including the arcades to the mid-periphery and increased signal disturbance within both maculae	Severely constricted field to all isopters 12e below threshold in both eyes <5 degrees	Severe widespread rod, cone, macular and RPE dysfunction with residual S cone response	c.226C>T c.1048C>G
**II**	F	White Caucasian	Night blindness (lifelong) (40)	69	Abnormal colour vision, poor contrast, mild photosensitivity Cataracts bilateral	6/36+1 RE 6/36 LE	Clumped pigmentary deposition extending from, and including the vascular arcades into the mid periphery, with far peripheral atrophy	Disrupted fovea, with thickened EZ and IZ	Increased AF at the maculae and at the arcades with decreased patchy AF in the areas of atrophy in the mid periphery	Partial ring scotoma to V4e stimulus with infratemporal sparing, central scotoma to III.4e in both eyes	PERG was almost extinguished. Both rods and cones were grossly affected, with a similarly reduced and delayed response to standard flash under both scotopic and photopic conditions The 30Hz flicker was markedly abnormal in the presence of a large S cone response.	c.119-2A>C Hom
**III**	F	White Caucasian	Nyctalopia, photophobia and photopsias (10)	16	Nyctalopia, photophobia, photopsias Cataract: no	6/5 RE 6/6 LE	Band of atrophy and white punctate dots in mid-peripheral retina circumferential to the macula	Central preservation of the retina	Increased AF at both maculae with a brighter ring internal to arcades, patchy reduction in signal mapping to the atrophic patches	Ring scotoma to 1.4e stimulus with central 10 degree island. Preserved fields bilaterally to large targets	Relatively well preserved PERG Extinguished rod-specific dim flash responses and reduced and delayed responses to standard flash under both photopic and scotopic conditions. The response to the 30 Hz flicker stimulus was abnormal.	c.119-2A>C c.639_640insT

AF, autofluorescence; ERG, electroretinogram; EOG, electrooculogram; EZ, ellipsoid zone; IZ, interdigitation zone; LE, left eye; OCT, optical coherence tomography; PERG, pattern electroretinogram; RE, right eye; VA, visual acuity.

## Supplementary Materials

The following are available online at https://www.mdpi.com/2073-4425/11/11/1288/s1, Table S1: Variants identified in *NR2E3* to date; Table S2: The thickness of the of ganglion cell layer–inner plexiform layer in the nasal, temporal and inferior retina. References [51,52,53,54,55,56,57,58,59,60,61,62,63,64,65,66,67,68,69,70,71,72,73,74,75,76,77,78,79,80,81,82,83,84,85,86,87,88,89,90,91,92,93,94,95,96,97] are cited in the Supplementary Materials.
