# Supplementary material for: Novel Pathogenic Sequence Variants in NR2E3 and Clinical Findings in Three Patients
Source: Genes (Basel). 2020 Oct 29;11(11):1288. doi: 10.3390/genes11111288 (PMC7716234; doi:10.3390/genes11111288)
Supplement: Supplementary file 1 [file genes-11-01288-s001.zip › genes-964680-supplementary.docx]

**Supplementary Table 1.** Variants identified in *NR2E3* to date. Numbering is based on transcript NM_014249.

| Variant |  |  | Effect | Phenotype | Reference |
| --- | --- | --- | --- | --- | --- |
| c.95G>A | Exon 1 | TGG>TAG | p.(Y32X) | RP (simplex) | [51] |
| c.119-3A>C | Intron 1 |  | Splicing | ESCS | [3,52] |
| c.119-2A>C | Intron 1 |  | Splicing | ESCS  GFS  CPRD | This report  [1,3,5,6,10,17,19,35,38,40,44–46,48,52–63] |
| c.119-1G>A | Intron 1 |  | Splicing | ESCS | [6] |
| c.119-57_166del | Intron 1 |  | Gross deletion | arRP | [63] |
| c.131C>A | Exon 2 | TCG>TAG | p.(S44X) | Helicoid subretinal fibrosis | [64] |
| c.133C>T | Exon 2 | TCG>TTG | p.(S44L) | arRP | [45] |
| c.142C>T | Exon 2 | CGC>TGC | p.(R48C) | ESCS | [39] |
| c.143_144delGCins25 | Exon2 |  | Complex rearrangement | arRP | [65] |
| c.145G>A | Exon 2 | GTG>ATG | p.(V49M) | ESCS | [3] |
| c.151G>A | Exon 2 | GGA>AGA | p.(G51R) | ESCS | [39] |
| c.166G>A | Exon 2 | GGG>AGG | p.(G56R) | adRP | [43,66–74] |
| c.166G>C | Exon 2 | GGG>CGG | p.(G56R) | adRP | [75] |
| c.188C>A | Exon 2 | GCC>GAC | p.(A63D) | adRP | [67] |
| c.191G>T | Exon 2 | TGC>TTC | p.(C64F) | arRP/RCD | [59] |
| c.del194-202del9 | Exon 2 | ACGGCTGCA>GCGGCTTCT | p.(N65_C67del) | GFS  ECSC | [17,44,48,76] |
| c.196C>T | Exon 2 | AAC>AAT | p.(N65N) | arRP | [45] |
| c.del196-201del6 | Exon 2 | GGCTGC>AGCGGC | p.(G66_C67del) | ESCS | [17] |
| c.del201-209del9 | Exon 2 | CAGCGGCTT>CTTCAAGAG | p.(C67_G69del) | ESCS | [1,38,52] |
| c.202A>G | Exon 2 | AGC>GGC | p.(S68G) | ESCS | [40] |
| c.211T>C | Exon 2 | TTC>CTC | p.(F71L) | ESCS | [52] |
| c.211-213del3 | Exon 2 | TTC>AAG | p.(F71del) | ESCS | [8] |
| c.219G>C | Exon 2 | AGG>AGC | p.(R73S) | ESCS | [46,60] |
| c.223G>A | Exon 2 | GTA>ATA | p.(V75I) | adRP | [77] |
| c.226G>A | Exon 2 | CGG>CAG | p.(R76Q) | ESCS | This report  [1,35,52] |
| c.227G>T | Exon 2 | CGG>CAG | p.(R76W) | ESCS | [1,63,78] |
| c.229C>T | Exon 2 | CGG>TGG | p.(R77W) | ESCS | [79] |
| c.242A>G | Exon 2 | TAC>TGC | p.(Y81C) | ESCS | [3] |
| c.245+8 C>T | Intron 2 |  | Splicing | arRP | [38,45] |
| c.248G>A | Exon 3 | TGC>TAC | p.(C83Y) | ESCS | [52,80] |
| c.263G>T | Exon 3 | GGG>GTG | p.(G88V) | ESCS | [48] |
| c.290G>A | Exon 3 | CGC>CAC | p.(R97H) | ESCS | [1,38,48] |
| c.305C>A | Exon 3 | GCC>GAC | p.(A102D) | ESCS | [10,19,48,52,60,61] |
| c.310C>T | Exon 3 | CGG>TGG | p.(R104W) | ESCS | [1,10,39,52] |
| c.311G>A | Exon 3 | CGG>CAG | p.(R104Q) | ESCS | [3,48,52,81] |
| c.328dupC | Exon 3 | CAG>CCA | p.(Q110fsX30) | ESCS | [82] |
| c.328C>T | Exon 3 | CAG>TAG | p.(Q110X) | arRP | [35] |
| c.349+5G>C | Intron 3 |  | Splicing | ESCS | [35,83] |
| c.352G>A | Exon 4 | GTG>ATG | p.(V118M) | adRP | [68] |
| c.353insC | Exon 4 | GTG>GTC | p.(V118fsX22) | ESCS | [48] |
| c.361G>A | Exon 4 | GAG>AAG | p.(E121K) | ESCS | [1] |
| c.364C>T | Exon 4 | CGC>TGC | p.(R122C) | arRP | [35,40,84] |
| c.373C>T | Exon 4 | CGA>TGA | p.(R125X) | ESCS | [40,42,79] |
| c.424C>T | Exon 4 | CGG>TGG | p.(R142W) | adRP | [85] |
| c.471del1 | Exon 4 | CCA>CCC | p.(P157fsX21) | ESCS | [48] |
| c.473G>A | Exon 4 | CGA>CAA | p.(R158Q) | arRP | [35] |
| c.481delA | Exon 4 | ACA>CAC | p.(T161fsX178) | ESCS | [3,18,48,60] |
| c.571+2T>C | Intron 4 |  | Splicing | arRP | [57] |
| c.639_640insT | Exon 5 | TCC>TCT | p.(P214SfsX39) | ESCS | This report |
| c.646G>A | Exon 5 | GGC>AGC | p.(G216S) | GFS | [52,86] |
| c.694G>A | Exon 5 | GTC>ATC | p.(V232I) | arRP | [87] |
| c.701G>C | Exon 5 | TGG>TCG | p.(W234S) | ESCS | [1,48] |
| c.724_725del | Exon 5 | TCC>CAG | p.(S242QfsX17) | ESCS | [88] |
| c.739C>T | Exon 5 | CGG>TGG | p.(R247W) | ESCS | [52] |
| c.747+1G>C | Intron 5 |  | Splicing | ESCS | [44] |
| c.755T>C | Exon 6 | CTG>CCG | p.(L252P) | ESCS | [29] |
| c.767C>A | Exon 6 | GCG>GAG | p.(A256E) | ESCS | [10,38,48,52] |
| c.767C>T | Exon 6 | GCG>GTG | p.(A256V) | ESCS | [53,55] |
| c.788T>C | Exon 6 | CTC>CCC | p.(L263P) | ESCS | [48] |
| c.790G>A | Exon 6 | GGG>AGG | p.(G264R) | ESCS | [58] |
| c.797T>A | Exon 6 | ATC>AAC | p.(I266N) | arRD | [61] |
| c.805_806del2 | Exon 6 | TGGTCTCTG>TGGTCGCCT | p.(S269SfsX70) | ESCS | [19] |
| c.827-843del | Exon 6 | CCTCTGCTGGCACCGCC>  CGAGGCCTCTGCTGCCG | p.(P276RfsX59) | ESCS | [38] |
| c.859G>A | Exon 6 | GGT>AGT | p.(G287S) | arRP | [45] |
| c.908T>C | Exon 6 | CTG>CCG | p.(L303P) | ESCS | [52] |
| c.919_920del2 | Exon 6 | CTAT>CTCT | p.(I307LfsX33) | ESCS | [20] |
| c.925C>G | Exon 6 | CGG>GGG | p.(R309G) | ESCS | [1,5,48] |
| c.926G>T | Exon 6 | CGG>CTG | p.(R309L) | GFS | [86] |
| c.926G>A | Exon6 | CGG>CAG | p.(R309Q) | ESCS | [52] |
| c.932G>A | Exon 6 | CGG>CAG | p.(R311Q) | ESCS  GFS | [1,3,5,8,15,19,35,38,40,41,43–45,47,48,52,55,60,61,63,76,89–92] |
| c.951del | Exon 6 | ACG>CGG | p.(T318RfsX6) | arRP | [93] |
| c.967dupA | Exon 6 | ATG>AAT | p.(M323NfsX17) | arRP | [94] |
| c.971 A>G | Exon 6 | AAG>AGG | p.(K324R) | arRP | [45] |
| c.994G>A | Exon 6 | GAG>AAG | p.(E332K) | ESCS | [10] |
| c.994G>T | Exon 6 | GAG>TAG | p.(E332X) | arRP | [62] |
| c.995-2A>C | Intron 6 |  | Splicing | arRP | [78] |
| c.1000C>G | Exon 7 | CGG>GGG | p.(R334G) | ESCS | [81] |
| c.1004G>A | Exon 7 | GGC>GAC | p.(G335D) | arRP | [35] |
| c.1007T>C | Exon 7 | CTG>CCG | p.(L336P) | ESCS | [48] |
| c.1018G>A | Exon 7 | GAG>AAG | p.(E340K) | ESCS | [95] |
| c.1025T>C | Exon 7 | GTA>GCA | p.(V342A) | ESCS | [10,52] |
| c.1025T>G | Exon 7 | GTA>GGA | p.(V342G) | arRP | [96] |
| c.1034_1038del | Exon 7 | TTGCAG>TGACCA | p.(K345X) | arRP | [45] |
| c.1048C>T | Exon 7 | CAA>TAA | p.(Q350X) | ESCS | [16] |
| c.1048C>G | Exon 7 | CAA>GAA | p.(Q350E) | ESCS | This report |
| c.1049A>G | Exon 7 | CAA>CGA | p.(Q350R) | ESCS | [8] |
| c.1057C>G | Exon 7 | CTG>GTG | p.(L353V) | ESCS | [48] |
| c.1095C>G | Exon 7 | CCC>CCG | p.(P365P) | ESCS | [48] |
| c.1101-1G>A | Intron 7 |  | Splicing | ESCS | [3] |
| c.1101-6C>G | Intron 7 |  | Splicing | arRP | [63] |
| c.1112T>G | Exon 8 | TTG>TGG | p.(L371W) | ESCS | [52,95] |
| c.1118T>C | Exon 8 | CTG>CCG | p.(L373P) | ESCS | [35,83] |
| c.1120C>T | Exon 8 | CTC>TTC | p.(L374F) | ESCS | [18] |
| c.1154G>C | Exon 8 | CGC>CCC | p.(R385P) | ESCS | [1] |
| c.1194delC | Exon 8 | CCA>CAA | p.(P399QfsX3) | ESCS | [10,52] |
| c.1217A>G | Exon 8 | GAT>GGT | p.(D406G) | GFS | [97] |
| c.1220T>A | Exon 8 | ATG>AAG | p.(M407K) | ESCS | [1,48] |
| c.1223delT | Exon 8 | TTC>TCA | p.(F408SfsX70) | arRP | [35] |
| c.1225A>G | Exon 8 | AAA>GAA | p.(K409E) | arRP | [35] |

**Supplementary Table 2.** The thickness of the of ganglion cell layer-inner plexiform layer in the nasal, temporal and inferior retina.

| Patient | Temporal thickness  (μm) | | Nasal thickness  (μm) | | Inferior thickness  (μm) | |
| --- | --- | --- | --- | --- | --- | --- |
|  | OD | OS | OD | OS | OD | OS |
| I | 23 | 23 | 8 | 13 | 13 | 10 |
| II | 16 | 18 | 22 | 23 | 8 | 18 |
| III | 23 | 28 | 18 | 24 | 21 | 18 |
